# Optic Nerve Stimulation System with Adaptive Wireless Powering and Data Telemetry †

**DOI:** 10.3390/mi8120368

**Published:** 2017-12-20

**Authors:** Xing Li, Yan Lu, Xiaodong Meng, Chi-Ying Tsui, Wing-Hung Ki

**Affiliations:** 1 Broadcom Corporation, Irvine, CA 92618, USA; 2 State Key Laboratory of Analog and Mixed-Signal VLSI, University of Macau, Macao, China; yanlu@umac.mo; 3 Department of Electronic and Computer Engineering, Hong Kong University of Science and Technology, Hong Kong, China; tommxd2010@gmail.com (X.M.); eetsui@ust.hk (C.-Y.T.); eeki@ust.hk (W.-H.K.)

**Keywords:** wireless power transfer (WPT), data telemetry, implantable medical device (IMD), neural stimulation

## Abstract

To treat retinal degenerative diseases, a transcorneal electrical stimulation-based system is proposed, which consists of an eye implant and an external component. The eye implant is wirelessly powered and controlled by the external component to generate the required bi-polar current pattern for transcorneal stimulation with an amplitude range of 5 μA to 320 μA, a frequency range of 10 Hz to 160 Hz and a duty ratio range of 2.5% to 20%. Power delivery control includes power boosting in preparation for stimulation, and normal power regulation that adapts to both coupling and load variations. Only one pair of coils is used for both the power link and the bi-directional data link. Except for the secondary coil, the eye implant is fully integrated on chip and is fabricated using UMC (United Microelectronics Corporation, Hsinchu, Taiwan) 0.13 μm complementary metal-oxide-semiconductor (CMOS) process with a size of 1.5 mm × 1.5 mm. The secondary coil is fabricated on a printed circuit board (PCB) with a diameter of only 4.4 mm. After coating with biocompatible silicone, the whole implant has dimensions of 6 mm in diameter with a thickness of less than 1 mm. The whole device can be put onto the sclera and beneath the eye’s conjunctiva. System functionality and electrical performance are demonstrated with measurement results.

## 1. Introduction

Retinal degenerative (RD) diseases are a clinically and genetically heterogeneous group of primary retinal abnormalities that cause degeneration of the photoreceptors (rods and cones) or the retinal pigment epithelium and lead to progressive loss of vision. Transcorneal electrical stimulation (TcES) is one of the therapies that promotes the survival of photoreceptors and preserves retinal functions [1]. Previous electrical stimulation on the eye was performed through an ERG-Jet contact lens electrode with the stimulation current provided by external equipment through a metal wire [2]. With this setup, the cornea has to be anesthetized for each treatment and is neither convenient nor portable for patients to use. Moreover, daily use of this TcES device has a high chance to induce tissue damage.

**Figure 1:**
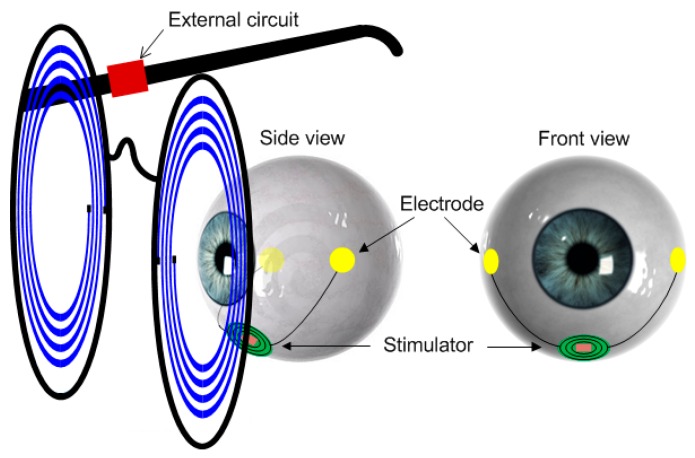
The transcorneal electrical stimulation (TcES) implant in the eye.

To enhance convenience and portability with a minimum invasive setup, one possible solution is to implant the whole stimulator underneath the eye. To reduce the side effects of tissue damage and invasive infection, and to reduce the complexity of the surgical operation so that it can be carried out in clinics instead of operation rooms, we propose to place this implantable stimulator beneath the eye’s conjunctiva but outside the eyeball on the sclera completely unexposed, as shown in Figure 1. The two electrodes are placed on the sclera at the two sides of the eyeball. In this paper, we present the design of such an on-sclera implant-based TcES system.

An implantable medical device (IMD) has to be small and thin for the comfort of the patients. Battery has to be eliminated due to its bulky size and limited lifetime [3]. Wireless power transfer using inductive coupling has been widely used for power transfer and data telemetry in IMD applications such as retinal prosthesis and neural stimulation and recording [4,5,6,7]. To achieve high power transfer efficiency and high data rate, multi-channels were proposed in [8,9,10,11] to transfer the power and data separately using two or more pairs of coils. In [12] and [13], 3-coil and 4-coil coupled power links were proposed, respectively, to enhance the coupling efficiency. For the TcES application, data rate does not need to be high and system volume has to be minimized. To reduce system volume, we propose that the power link and the data link should share only one pair of coils. On-chip coil [14,15] could be used to further reduce the size, but the power transfer efficiency and the voltage gain would be significantly deteriorated due to poor quality factor and weak coupling with the primary coil. Hence, off-chip coil is adopted.

The human tissue specific absorption rate (SAR) increases with the coupling frequency. Therefore, inductive power links with 13.56 MHz or lower ISM bands are commonly used [16,17]. However, lower coupling frequency means larger matching and filtering capacitors that lead to a larger implant size. In [15], 40.68 MHz in the ISM band was proposed as a reasonable frequency in achieving good power transfer efficiency for the power link of IMDs. When compared with using 13.56 MHz, the matching and filtering capacitors at 40.68 MHz are reduced by 9 and 3 times, respectively. Hence, 40.68 MHz was finally chosen as the power coupling frequency.

This paper is organized as follows. Section 2 gives an overview of the system and the design of the power and data links, including control methods for power boosting and regulation. Detailed circuit implementation is presented in Section 3. Off-chip circuitry and coil design are discussed in Section 4. Measurement results of the fabricated chip are given in Section 5, and conclusions are drawn in Section 6.

## 2. System Overview

### 2.1. System Functionality

**Figure 2:**
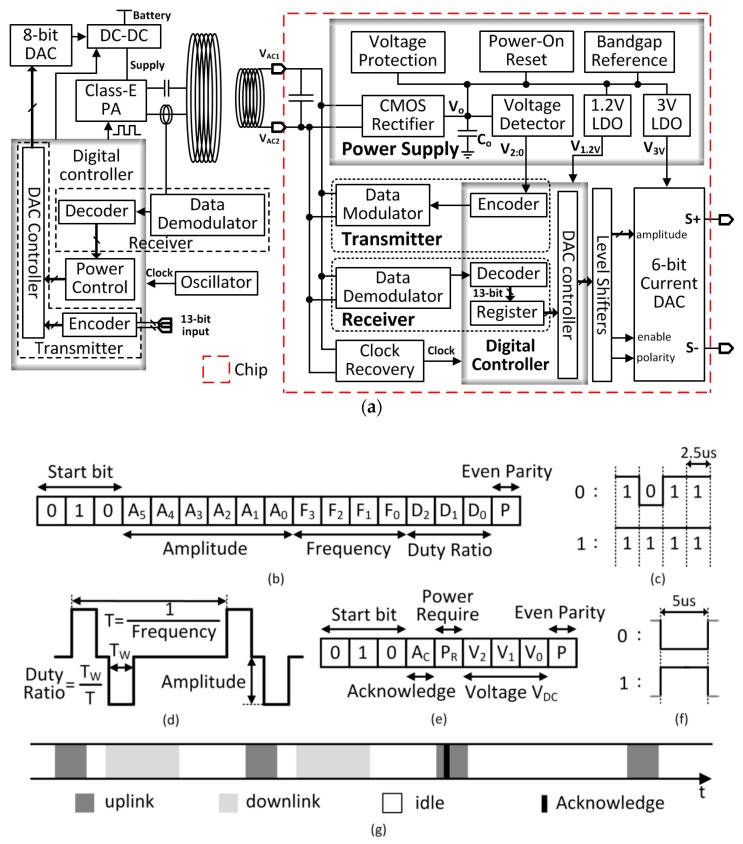
(**a**) The whole TcES system diagram (**b**) Downlink data package (**c**) Downlink data coding (**d**) Retina stimulation current (**e**) Uplink data package (**f**) Uplink data coding (**g**) Time multiplexing of the uplink and the downlink.

The TcES system is shown in Figure 2a [18]. It consists of an external control unit and an internal implant unit. Except for the secondary coil, the implant unit is fully integrated on-chip and is shown in the dash box in Figure 2a. The internal implant unit consists of the power supply module, the data telemetry module, the digital controller and the stimulation unit. The inductively coupled AC power on the secondary coil is converted into DC power by the rectifier and stored in the on-chip filtering capacitor at V_DC_. A 1.2-V low dropout regulator (LDO) and a 3-V LDO powered by V_DC_ are used to supply power to the digital controller and the stimulation unit, respectively. The voltage detector monitors V_DC_ for controlling the power. Peripheral circuits include the voltage protection circuit and the power-on reset for over-voltage protection and initialization during startup, respectively. The bandgap voltage reference generates the reference voltages for the 1.2-V LDO and the 3-V LDO, and also the bias currents for the stimulation unit. The data telemetry circuitry consists of a transmitter that includes an encoder and a data modulator, and a receiver that includes a demodulator and a decoder. The clock for the digital block is recovered from the power coupling frequency by a clock recovery circuit.

The external control unit consists of the power transmission circuitry and the data telemetry circuitry. The DC-DC buck converter supplies power to the Class-E power amplifier that drives the series-resonant primary coil. An 8-bit digital-to-analog converter (DAC) is used to adjust the DC-DC output voltage to control the transmission power of the primary coil. A detection coil is used to receive the back-scattered data transmitted from the internal implant unit, and a digital controller is used to encode and decode the data.

For this TcES system, the power link and the data link share the same pair of coils. The output voltage of the rectifier V_DC_ changes when the loading changes, as well as when the distance of the coils or their relative alignment changes. Adaptive power control is needed to dynamically adjust the emitted power to achieve a stable rectified DC voltage. It is divided into power regulation and power boosting. For power regulation, the voltage detector senses V_DC_ periodically and the value is transmitted back to the external control unit through backscattering. By comparing with a reference voltage, the output of the DC-DC converter is adjusted to change the transmission power accordingly. Power boosting is activated to increase the transmission power prior to the scheduled task of electrical stimulation. With these proposed power control strategies, the rectified DC output is well regulated and achieves better immunity to coupling and loading variations.

The implant unit generates a bi-polar current waveform for retina stimulation. For different patients or different therapy stages of the same patient, the stimulation profiles (current amplitude, stimulation frequency and duty ratio of current pulse) are different. The stimulation profile is programmed by the external control unit and sent through the data link. The digital controller reads the 13-bit configuration data, codes it, modulates it with amplitude shift keying (ASK) and then transmits it to the internal implant unit. The implant circuitry demodulates and decodes the 13-bit data, and stores it in a register that sets the DAC output with the stimulation profile. The current amplitude can be programmed to range from 5 μA to 320 μA, the stimulation frequency from 10 Hz to 160 Hz, and the current pulse duty ratio from 2.5% to 20%.

### 2.2. The Data Link

The downlink data package is shown in Figure 2b. It contains the DAC controller configuration information. The 13 bits are organized as shown in Figure 2c. The start bits “010” signifies the start of a data package, which also helps improve the bit error rate (BER). The current amplitude control bits A_5:0_ corresponds to 5 μA to 320 μA with 6-bit quantization; the frequency control bits F_3:0_ corresponds to 10 Hz to 160 Hz with 4-bit quantization; and the duty ratio control bits D_2:0_ corresponds to 2.5% to 20% with 3-bit quantization. The final bit P is the even-parity check bit. ASK modulation is used for the downlink. In the idle state, the idle bit is “1”, and power but not data is transferred. If the data “1” is transmitted, power transfer is not affected. However, if the data “0” is transmitted, power transfer is reduced abruptly to generate a negative pulse, and the implant unit may not obtain enough power. Due to the limited bandwidth of both the primary and secondary *LC* tanks, high data rate may not be easily achieved. The situation gets worse as the downlink data package has 17 bits in total, and the data package time-slot is quite long. In the worst case of transmitting a long chain of “0” s, the rectifier output would drop as the on-chip filtering capacitor is not too large. The solution is to use the coding scheme as shown in Figure 2b: “0” is coded as “1011”; and “1” is coded as “1111”. Therefore, even a long chain of data “0” s are transmitted the implant unit could get 75% of full power, which is designed to be adequate. An additional benefit is the relaxed requirement of the internal decoder. The data are decoded as “0” s and “1” s with and without negative pulses, respectively. Both pulse width and pulse phase are not required to be accurate. With this coding scheme, the length of the downlink data package is 170 μs.

The 9-bit uplink data package that is sent to the external control unit is shown in Figure 2e. The first 3 bits are the “010” start bits. The 4th bit A_C_ is the active-low acknowledgement bit. The 5th bit P_R_ is the power requirement bit for power boosting, which will be explained later. The next 3 bits V_2:0_ indicate the quantized rectifier output voltage for power regulation; and the last bit P is the even-parity check bit. The uplink is based on backscattering that also uses ASK modulation. The uplink data transmission affects the power received, but as the data package has only 9 bits, the duration is much shorter. To balance the package duration and its effect on the received power, no special coding is used. The length of the uplink data package is 45 μs.

Both the downlink and the uplink are ASK modulated that share the same coupling channel, and they cannot work simultaneously. Time multiplexing is used as shown in Figure 2f. The uplink data package is sent to the external control unit every 500 μs. The downlink data transmission interleaves with the uplink, but it only transmits data when there is a new configuration input data. When the new data is transmitted, the acknowledgement bit A_C_ will be checked in the next uplink data package. If there is no acknowledgement, this new data will be retransmitted until the external control unit receives A_C_ from the internal implant unit.

### 2.3. The Power Link

**Figure 3:**
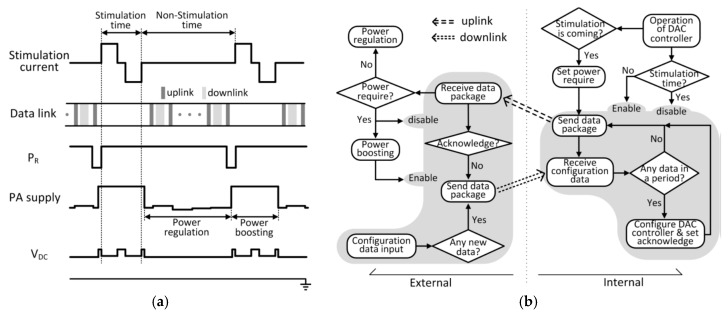
(**a**) Power control waveform (**b**) System state diagram.

Power is wirelessly delivered from the external control unit to the internal implant unit using near-field coupling. For retina stimulation, the stimulation current is issued periodically as shown in Figure 3a. During the non-stimulation time, the loading of the implant is light as it is only the total quiescent power of each block. During the stimulation time, the loading is heavy by issuing the stimulation current from the current DAC. Hence, power delivery is divided into the power-boosting phase and the power-regulation phase that are responsible for the stimulation time and the non-stimulation time, respectively.

The distance and the alignment of the two coupling coils may vary that affect the coupling coefficient and thus the power received. During the non-stimulation time, the power-regulation phase is activated, and the rectifier output V_DC_ is periodically sensed and sent to the external control unit to adjust the transmitter power so that V_DC_ could be regulated to 3.5 V. The movement of the human body is on the order of milliseconds, and so an uplink data package rate of 2 kHz is used.

During the stimulation time, the small on-chip filtering capacitor is drained to provide the stimulation current, and V_DC_ will drop sharply. As the uplink package rate is low, it cannot adapt to this fast loading variation. To cater for this scheduled loading variation, the power requirement bit P_R_ is set active (active low) just prior to the stimulation time, and is sent to the external control unit to activate the power-boosting phase, such that even V_DC_ drops, it is still high enough for the downstream circuits to operate properly. During the stimulation time, the data link is disabled to avoid interference.

In the power-boosting phase, the transmitting power has to be increased to cover the whole stimulation time with adequate margin. For different coupling scenarios and stimulation profiles, the amount of increase in power is different (instead of using the maximum power) to avoid excessive radio frequency (RF) power that will be absorbed by the human body. Calibration is performed during the assembly phase of the implant unit. Load variation is related to the stimulation current amplitude, and coupling variation is related to the steady state PA voltage V_DD,PA_. By sweeping the above parameters for all possible cases and recording the needed power-boosting level to ensure adequate power to cover the whole stimulation time with margin, a look-up table is constructed to store the data of power increase required for normal operation.

### 2.4. The System State Diagram

The TcES system state diagram is shown in Figure 3b. The shaded parts deal solely with the data link; and the non-shaded parts deal with the power link that also depends on the data link. The startup phase of the system will be discussed in Section 2.5.

Let us consider the system operation after startup during the non-stimulation time, that is, during the power-regulation phase. The external control unit and the internal implant unit exchange roles as master and salve every 500 μs. Consider that the configuration data is inputted (through programming the external control unit), and when the external control unit resumes the master role it is then sent across the link. If the internal implant unit receives the data, it will be used to configure the DAC controller and the acknowledgement bit A_C_ will be set active low to be sent back to the external control unit in the next data package. After the data package is sent, A_C_ will then be reset. If there is no configuration data received within this period, the internal implant unit just sends back the uplink package that consists of the digitized rectifier output voltage V_DC_, so that the external control unit could adjust the transmitting power to regulate V_DC_. After receiving the data package, the external control unit becomes the master. If there is a new configuration data needed to be transmitted and there is no acknowledgement from the internal unit, the data will be sent and then the external unit shifts to be slave again waiting to receive data. If the above condition is not satisfied, no data will be sent and the external unit becomes slave after 500 μs.

For a certain stimulation profile, the internal implant unit knows the period of stimulation and knows exactly when the next stimulation slot will arrive. Just before the next scheduled stimulation time slot, the power-boosting phase has to be activated. Hence, when the internal implant unit becomes the master, the power require bit P_R_ is set active low and sent to the external control unit to increase the transmitting power according to the look-up table. During the stimulation time, the data telemetry block of the internal implant unit is disabled. Note that the data telemetry block of the external control unit is disabled when the instruction for power boosting is received; and it is enabled after power boosting is completed.

### 2.5. The Startup

The power link relies on the data link to determine the transmitting power level, but the data link can function only if there is sufficient received power. During startup, the internal implant unit has no stored energy. To solve this problem, during the TcES system startup, the external control unit will issue a very high power to jump-start the internal implant unit. Once the data link is established, the transmitting power will decrease slowly. Eventually, the transmitting power will be controlled according to the power-regulation and the power-boosting mechanisms as previously discussed. The startup procedure will be repeated if the first trial fails. However, if it fails repeatedly due to weak coupling or misalignment between the coupling coils, the external control unit will be shut down for protection.

## 3. On-Chip Circuit Implementation

### 3.1. CMOS Rectifier and Voltage Protection

**Figure 4:**
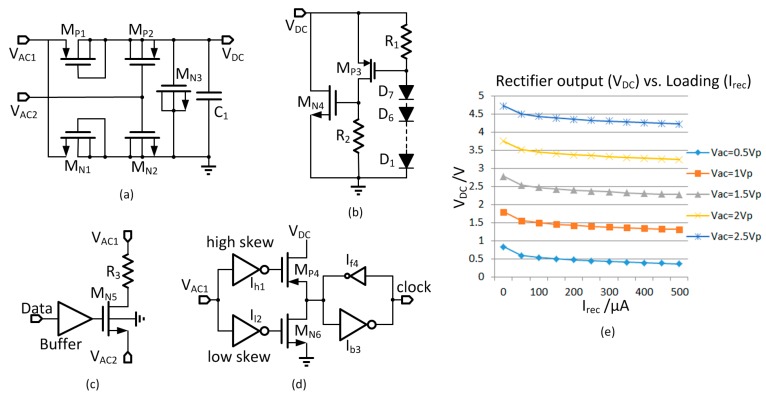
(**a**) Complementary metal-oxide-semiconductor (CMOS) rectifier; (**b**) Voltage protection circuit; (**c**) Implant modulator; (**d**) Clock recovery circuit; (**e**) Simulated rectifier output vs. loading.

Power is coupled from the primary coil to the secondary coil, and AC voltage is developed between V_AC1_ and V_AC2_, the two terminals across the secondary LC tank. A rectifier plus voltage doubler as shown in Figure 4a is used to convert the AC voltage to a DC voltage to power up the internal implant unit. The transistors M_N1_ and M_P1_ with low threshold voltages of 0.25 V are diode-connected; the transistors M_N2_, M_N3_ and M_P2_ are connected as metal oxide semiconductor (MOS) capacitors, and C_1_ is composed of both metal-insulator-metal (MIM) and MOM capacitors to maximize the capacitance density. The diode M_P1_ and the capacitor M_P2_ constitute the first rectifier for V_AC1_ > V_AC2_; and the diode M_N1_ and the capacitor M_N2_ constitute the second rectifier for V_AC2_ > V_AC1_; and the two rectifiers are stacked up as a voltage doubler with filtering capacitors M_N3_ and C_1_. The doubler improves the voltage gain from the PA supply voltage to the rectifier output and relaxes the design of the coupling coils and the supply voltage requirement of the external control unit. In the steady state, V_AC2_ is a DC voltage of V_DC_/2, while V_AC1_ swings between around −0.3 V and V_DC_ + 0.3 V. In one cycle, V_AC1_ charges the first and the second rectifier in the positive and the negative phase, respectively, and the ripple frequency of V_DC_ is 81.36 MHz that is double of the power carrier frequency. Figure 4e shows the simulated V_DC_ vs. rectifier loading current (I_rec_).

In the steady state, the output voltage of the rectifier V_DC_ is regulated to around 3.5 V during the power-regulation phase. However, during startup, V_DC_ could even be higher than the breakdown voltage of the process. Therefore, a protection circuit of 7 diodes in series (Figure 4b) is added at the output of the rectifier to clamp V_DC_ to within around 4.5 V. When V_DC_ is higher than 4.5 V, M_P3_ is turned on, which then turns on M_N4_ to discharge the filtering capacitors across V_DC_ to prevent it from getting higher.

### 3.2. Implant Modulator and Clock Recovery

The data modulator at the implant uses backscattering based on load shift. As shown in Figure 4c, a switch M_N5_ is used to shunt the resistor R_3_ with the secondary *LC* tank. In the non-stimulation time, the equivalent loading resistance across the secondary coil is around 12.5 kΩ. During backscattering, the input data controls whether R_3_ should be shunt or not. Effectiveness of backscattering depends on the amount of load shift, but if R_3_ is small, too much power would be dissipated, and the voltage on the secondary LC tank may be reduced too much that affects the clock recovery circuit. After careful consideration, R_3_ is designed to be 500 Ω. The threshold voltage of M_N5_ is 0.5 V, and it can be completely turned on and off by V_DC_ and 0 V, respectively.

The clock recovery circuit is shown in Figure 4d. It recovers the clock from the secondary resonant *LC* tank to drive the digital controller. The high skew and low skew inverters I_h1_ and I_l2_ together with M_P4_ and M_N6_ form a Schmitt trigger comparator for noise rejection. When V_AC1_ goes high, M_N6_ is turned off first, and after the designed dead-time M_P4_ is turned on. Similarly, when V_AC1_ goes low, M_P4_ is turned off first and after the dead time, M_N6_ is turned on. The transistors of I_h1_ and I_l2_ are very small to reduce the short-circuit current. The isolation inverter I_b3_ and the tiny feedback inverter I_f4_ are used to hold the voltage level during the dead-time durations.

### 3.3. V_DC_ Detector

**Figure 5:**
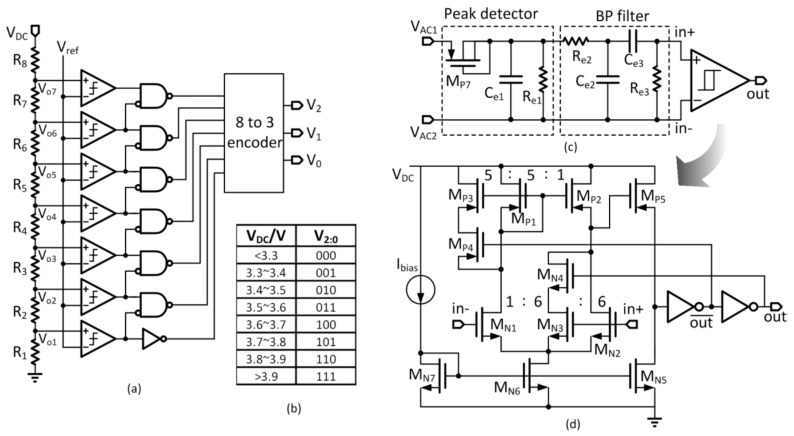
(**a**) Voltage detector (3-bit flash analog-to-digital converter (ADC)) (**b**) Quantization code (**c**) Internal demodulator (**d**) Schmitt trigger comparator.

The rectifier output voltage V_DC_ is quantized into a digital word for uplink transmission. Quantization noise could be reduced by using more quantization bits, at the expense of a longer uplink data package. We decided to use 3 bits, and V_DC_ is quantized into V_2:0_ by a 3-bit flash analog-to-digital converter (ADC) as shown in Figure 5a. As V_DC_ is regulated to around 3.5 V in the power-regulation phase, to make full use of the 3 bits the quantization range is designed to be from 3.2 V to 4.0 V with a resolution of 0.1 V. The quantization code is shown in Figure 5b. The bandgap reference generates the reference voltage V_ref_ of 1.2 V. The flash ADC consists of a resister ladder with 7 outputs V_o1–o7_ that are compared to V_ref_; and the outputs of the comparators are encoded into the 3-bit word V_2:0_. The resistor ladder has a total resistance of 1 MΩ.

### 3.4. Implant Demodulator

The downlink data is ASK modulated, and an envelope detector is used as the implant demodulator as shown in Figure 5c. It consists of a peak detector, a band-pass (BP) filter and a Schmitt trigger comparator. The peak detector is a half-wave rectifier driving the capacitor C_e1_ that filters out most of the carrier frequency component. The BP filter in cascade has a center frequency of 400 kHz and is used to capture the clean data to be sent to the Schmitt trigger comparator for quantization. In the steady state, V_AC2_ is a DC voltage of V_DC_/2 and is connected to the negative input terminal in- of the Schmitt trigger comparator. The data signals are single-ended that drives the positive input terminal in+. The Schmitt trigger comparator has two negative thresholds: −0.1 V and −0.3 V. In the idle state, in+ is almost equal to in- and the output is “High”. When “1” is transmitted, it is the same as the idle state. When “0” is transmitted, if the negative pulse goes below −0.3 V, the output will be triggered to “Low”. Negative pulses due to noise or power regulation with amplitudes lower than 0.3 V will be rejected. The 0.2 V threshold window is used to avoid multiple switching in one negative pulse.

The Schmitt trigger comparator is shown in Figure 5d. Both the input transistor pair and the current mirror are designed not to be matched to generate different input offset voltages that serve as the negative threshold voltages. When the output is “low”, M_N4_ and M_P4_ are turned off. The mismatch between M_N1_ and M_N2_, and the mismatch between M_P1_ and M_P2_ together result in a negative triggering threshold of around −0.1 V. When the output is “high”, M_N4_ and M_P4_ are turned on. The mismatch between M_N1_ and M_N2_ + M_N3_ and the mismatch between M_P2_ and M_P1_ + M_P3_ result in another negative triggering threshold of around −0.3 V.

### 3.5. Symmetrically Matched Bandgap Reference

**Figure 6:**
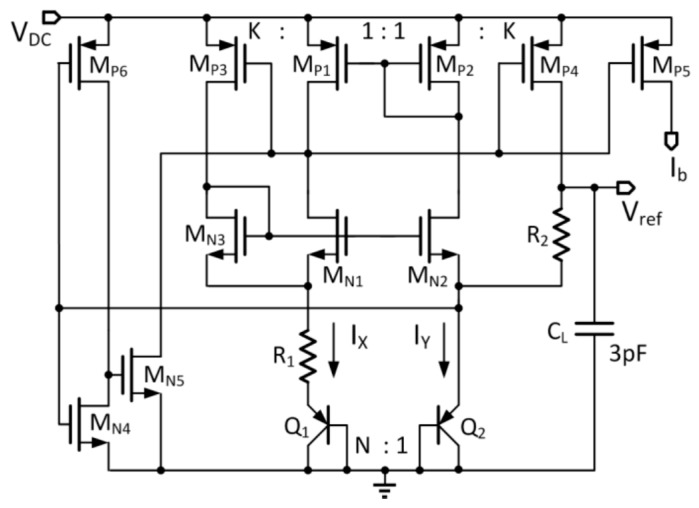
The two-branch self-biased symmetrically matched bandgap reference.

To minimize the quiescent current, a symmetrically matched (SM) bandgap reference, as shown in Figure 6, is used [19]. Two transistors are symmetrically matched if they have essentially the same V_gs_ (MOSFET gate-to-source voltage) and V_ds_ (MOSFET drain-to-source voltage). In this topology, only two branches are needed to generate the bandgap voltage V_ref_ (around 1.2 V). The currents I_X_ and I_Y_ are well matched with SM transistor-pairs (M_N1_, M_N2_) and (M_P1_, M_P2_). If V_gs3_ ≈ V_R2_, then M_P3_ and M_P4_ are also symmetrically matched. In this way, the error factor of the mismatch current is reduced. The startup circuit consists of M_N4_, M_N5_ and M_P6_. The reference voltage is given by

(1) $$V_{\mathrm{ref}}=V_{\mathrm{EB}2}+\frac{R_{2}}{R_{1}}\frac{K}{K+1}\ln\left(N\right)\times V_{T}$$

where N and K are the size ratios of Q_1_:Q_2_ and M_P3_:M_P1_, respectively. In this design, K = 3 and N = 8 for matching considerations.

The total current of the reference is less than 2 μA, excluding the bias currents provided to other circuit blocks. A 3 pF n-type metal-oxide-semiconductor (NMOS) load capacitor C_L_ is connected to V_ref_ to improve its power supply rejection (PSR) at around 80 MHz.

### 3.6. Linear Regulators

**Figure 7:**
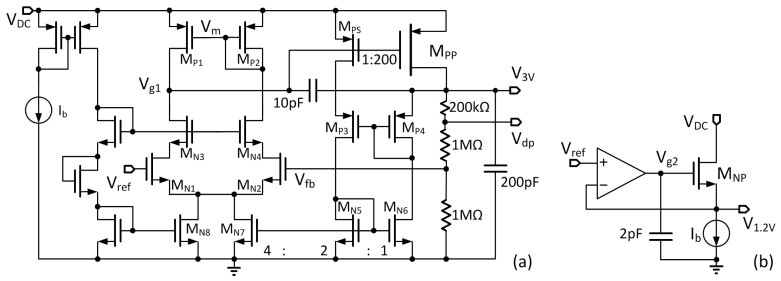
(**a**) The 3-V low dropout regulator (LDO) for electrical stimulation (ES) generator and (**b**) the 1.2-V LDO for digital blocks.

The wirelessly transferred power is not very steady. The rectifier output voltage V_DC_ has ripples at 81.36 MHz, and fluctuates when there is relative movement between the coupling coils. Thus, a regulator is needed to regulate V_DC_ to a predefined constant voltage for the load. Two regulators are implemented in the implant unit as shown in Figure 7a,b, one for generating a 3 V supply for the electrical stimulation (ES) generator, and the other for generating a 1.2 V for the digital controller. The 3-V LDO is used to provide the stimulation current pulses that switch between zero and the maximum current during stimulation. A sensing metal-oxide-semiconductor field-effect transistor (MOSFET) M_PS_ is used to sense the output current, and then the bias current of the error amplifier is made adaptive to the output current with an additional bias current provided by M_N7_ [20]. The loop is stabilized by Miller compensation with a 10 pF MIM capacitor. The quiescent current of the 3-V LDO is less than 3 μA at light load, and is 21 μA when the load current is 1 mA. A 200 pF NMOS capacitor is connected to the output for PSR improvement.

The 1.2-V LDO shown in Figure 7b is a source follower that has low output impedance and fast response. This LDO consumes a quiescent current of 2 μA. A 2 pF decoupling capacitor is connected to V_g2_ to improve its PSR performance.

### 3.7. Electrical Stimulation Current Generator

**Figure 8:**
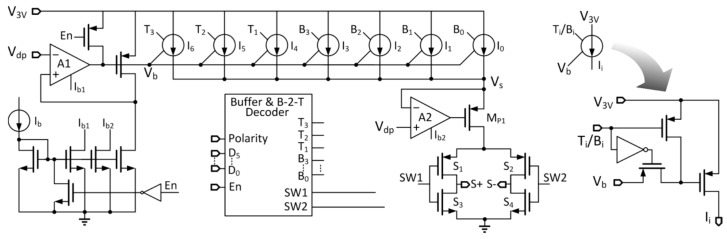
6-bit current digital-to-analog converter (DAC) for ES.

A high output impedance 6-bit current digital-to-analog converter (DAC) is used to generate the bi-polar stimulation current and the schematic is shown in Figure 8. Although bi-polar current pulses are used, charge neutrality has to be ensured to avoid damaging the human tissue. To guarantee charge neutrality, usually a large DC blocking capacitor is added to the electrodes [21]. However, it is not easy to integrate large on-chip capacitors, and precise charge-balanced biphasic current stimulator and single-channel bipolar current stimulator have been proposed in the literature [21,22,23].

In Figure 8, the stimulation current pulse amplitude is controlled by a 6-bit digital input from the digital controller. The current enable signal is “En” and the current direction indicator is “Polarity”. When “En” = 1 and “Polarity” = 1, “SW1” = 0 and “SW2” = 1, and the stimulation current will go out from S+ and come in from S−. If “En” = 1 and “Polarity” = 0, then “SW1” = 1 and “SW2” = 0, and the current flows in from the opposite direction. When “En” = 0, “SW1” = “SW2” = 1, there will be no output current and both S+ and S− will be shorted to the ground. As the positive and the negative current pulses are generated by the same current source, the residual DC current only depends on the difference of their durations. As their durations are controlled by the 40 MHz recovered clock, the timing mismatch is minimized. Moreover, in every cycle, S+ and S− will be shorted in non-stimulation time to discharge the residual accumulated charge, and charge neutrality is ensured.

With reference to Figure 7a, V_dp_ is designed such that V_3V_ − V_dp_ = 0.3 V. Now, the amplifier A1 generates V_b_ that biases the current sources I_0~6_ and the amplifier A2 generates V_s_ that is equal to V_dp_, to guarantee that the current sources will operate in the saturation region. Meanwhile, A2 and M_P1_ act as a gain-boosting stage of the DAC output, which helps improve the output impedance.

To improve the linearity and reduce the glitches, the two most significant bits are implemented in thermometer code [22]. The output current could be adjusted from 5 μA to 320 μA with a resolution of 5 μA/step; the duty ratio can be adjusted from 2.5% to 20% with a resolution of 2.5%/step; and the stimulation pulse frequency can be set in the range of 10 Hz to 160 Hz with a resolution of 10 Hz/step by the external controller.

## 4. Implementation of Off-Chip Components

### 4.1. Primary Coil, Secondary Coil and Detection Coil

**Figure 9:**
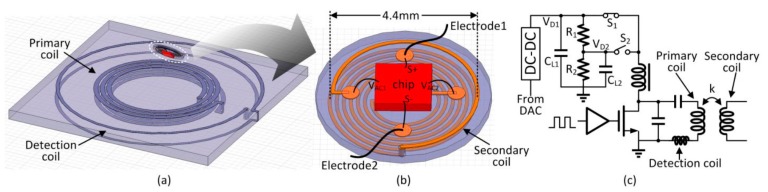
(**a**) The primary and detection coil (**b**) The secondary coil (**c**) The external circuitry.

Power delivery from the external control unit to the internal implant unit depends on the coupling of the primary and the secondary coils. Good link efficiency requires high quality factors for both coils. As shown in Figure 9a,b, all the three coils are two-layer designs printed on PCBs to have larger inductance within the limited area. The primary coil and the detection coil have outer diameters of 2 cm and 3 cm, respectively, and both are designed on the same PCB. The diameter of the PCB of the implant unit is 5 mm with a thickness of 0.3 mm. The outer diameter of the secondary coil is only 4.4 mm, and based on HFSS simulation, the inductance of the secondary coil is 170 nH and resonates with a 90 pF on-chip tuning capacitor at 40.68 MHz.

### 4.2. Class-E Power Amplifier and Its Power Supply

The primary coil is driven by a Class-E power amplifier with a supply voltage generated by a DC-DC converter that is controlled by an 8-bit DAC. Since power regulation is mainly adapted to slow coupling variation and power boosting is scheduled in advance for fast loading variation, the tracking speed of the DC-DC converter does not need to be very fast. However, if the ASK data modulation for the downlink data transmission is directly implemented by changing the supply voltage of the DC-DC converter, the tracking speed requirement will be much higher. In “0” transmission, the PA’s supply has a negative voltage pulse of only 2.5 μs that is difficult to be generated by the DC-DC converter. Instead, the narrow negative pulse is generated using the circuit shown in Figure 9c. Through a resistor string, a lower voltage V_D2_ is generated from the higher DC-DC output voltage of V_D1_. For most of the time, S_1_ is turned on and V_D1_ supplies power to the PA. Only in “0” transmission that S_2_ is turned on and the capacitor C_L2_ provides V_D2_ to generate the negative pulse. As the downlink is not frequently activated, the power overhead of sending the data is minimal. The resistor string is designed to be large enough such that its quiescent current is less than 10 μA, which is negligible.

## 5. Experimental Results

**Figure 10:**
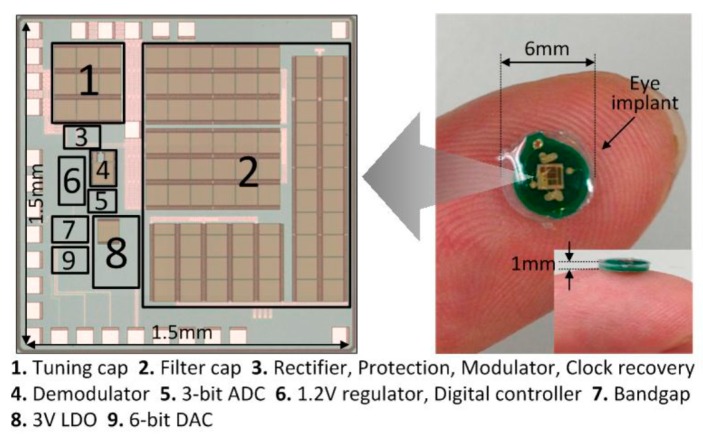
The die photo and the eye implant.

**Figure 11:**
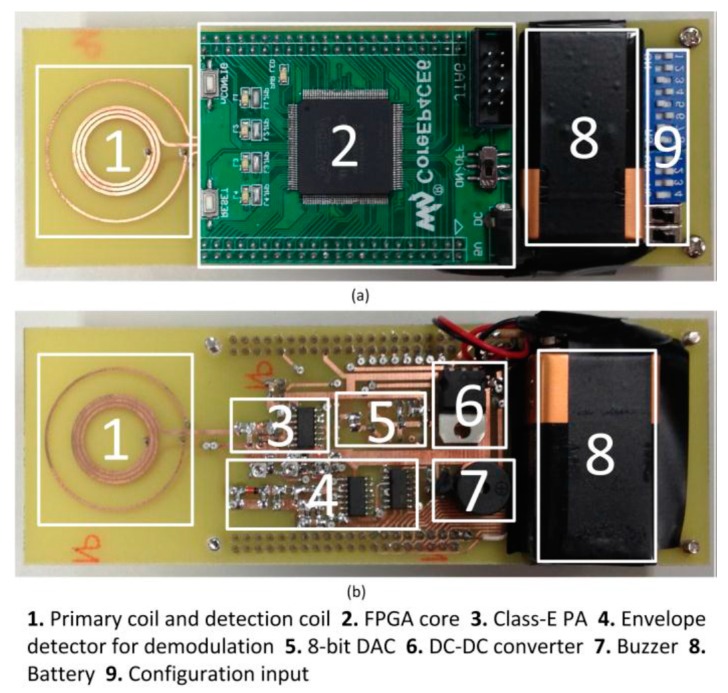
The external control unit (**a**) top view (**b**) bottom view.

The internal implant unit is fabricated using UMC 0.13 μm CMOS process. The die photo is shown in Figure 10 and the overall chip area is 2.25 mm^2^ and the thickness is 0.2 mm. The filtering capacitor consists of stacked MOS, MIM and MOM capacitors to maximize the capacitance density. The tuning capacitor is made of MIM capacitors, and the bottom plate is connected to V_AC2_ that is clamped at around V_DC_/2. To save space, the MOS and MOM capacitors underneath the tuning capacitor are also used as part of the filtering capacitor, and simulations showed no observable effect on the tuning circuit. The chip is glued on the secondary PCB and bonded directly to the coil as shown in Figure 9b. The whole implant unit is then encapsulated by biocompatible silicone. The overall thickness of this implant device is controlled to be less than 1 mm with an outer diameter of 6 mm, as shown in Figure 10. The whole eye implant is much smaller than a contact lens and can be put beneath the eye’s conjunctiva easily. The external control unit is implemented using discrete components and a field-programmable gate array (FPGA) as shown in Figure 11. A buzzer is added. When the distance of the two coils is too far apart, the buzzer will beep to alarm the patient to adjust the primary coil to make it closer to the eye implant. This external control unit will be integrated into a single chip in the future work.

**Figure 12:**
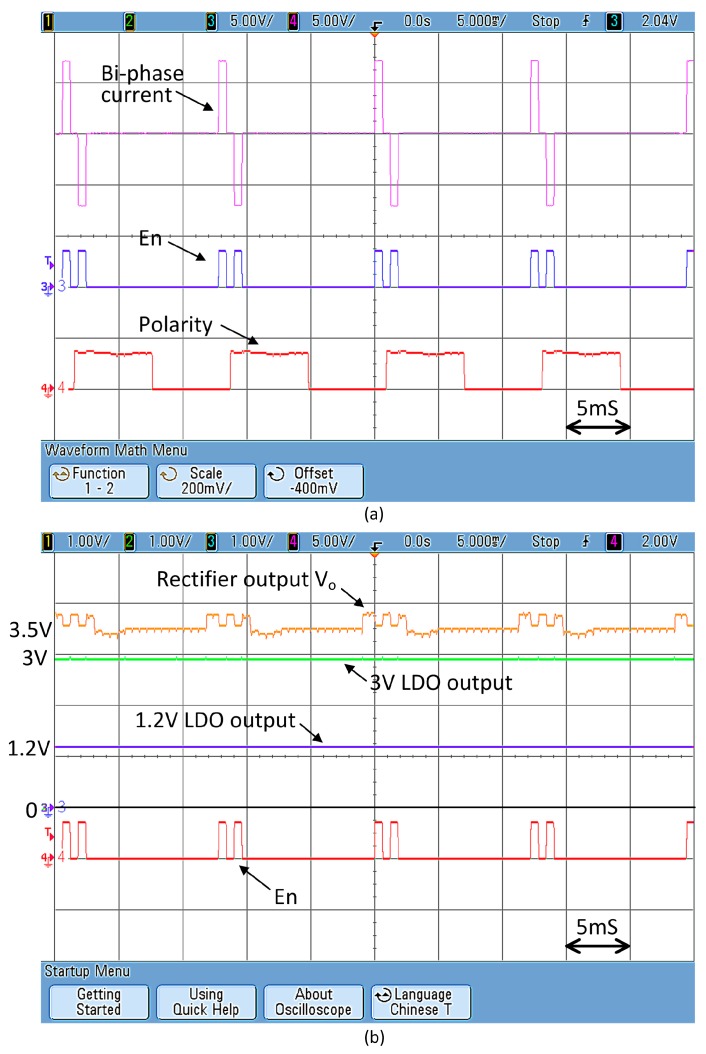
Measured operation waveform of (**a**) 6-bit current DAC (**b**) rectifier, 3 V LDO and 1.2 V LDO.

Figure 12a shows the measured waveform of the 6-bit current DAC with a 5 kΩ output load resistor. There is stimulation current when “En” = 1. The signal “Polarity” is a periodic square wave that changes the current direction. In Figure 12b, it is shown that the output voltage of the rectifier V_DC_ is not very steady. The voltage ripple consists of two components: the first one is the large voltage ripple of around 400 mV that has a frequency of lower than 1 kHz due to power boosting; and the second is the smaller voltage ripple of 50 mV at 80 MHz due to backscattering. After filtering by the 3-V LDO and the 1.2-V LDO, both ripple components are significantly attenuated. The stable 3 V and 1.2 V outputs are then used to supply power to the DAC and the digital controller, respectively.

**Figure 13:**
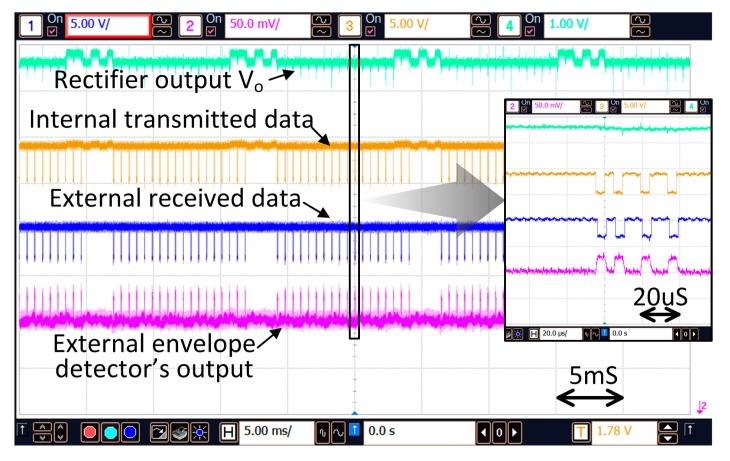
V_DC_ regulation by the backscattered data.

**Figure 14:**
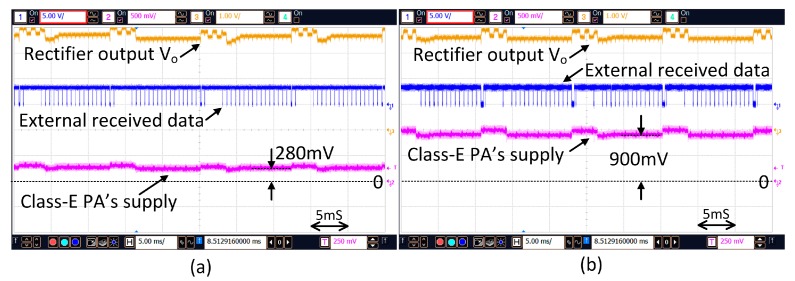
V_DC_ regulation under the two coils’ distance of (**a**) 4 mm (**b**) 1.3 cm.

Figure 13 shows the action of power regulation through data backscattering in the non-stimulation time. V_DC_ is periodically detected and the equivalent digital value is backscattered to the external control unit. The external control unit then adjusts the transmitting power such that V_DC_ is regulated to the predefined value of 3.5 V. When the distance between the two coils changes the supply voltage of the PA will be adjusted adaptively. As shown in Figure 14, when the distance changes from 4 mm to 13 mm, the supply voltage of the PA is increased from 280 mV to 900 mV such that V_DC_ is maintained at around 3.5 V.

**Figure 15:**
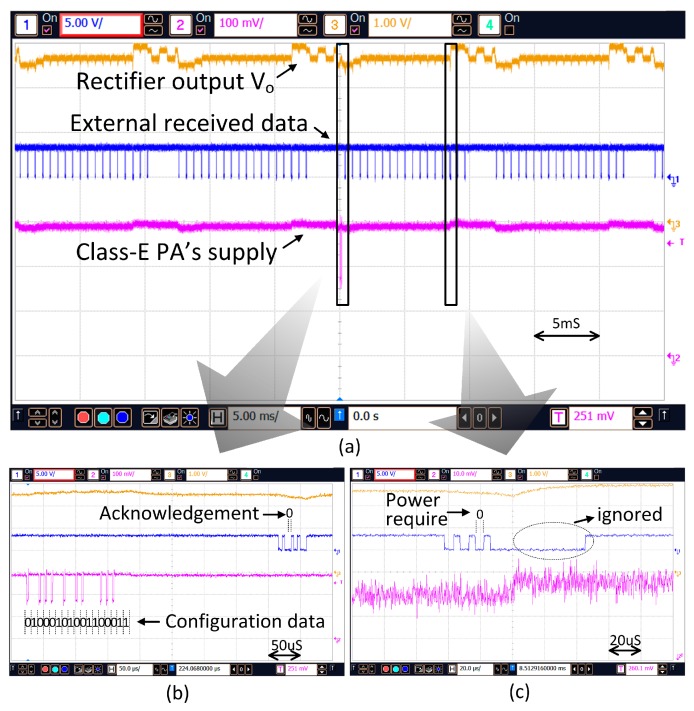
(**a**) Configuration data transmission and the power boosting (**b**) Configuration data transmission and acknowledgement (**c**) the power boosting for stimulation.

Figure 15a shows the transmitted configuration data and the accompanied power boosting during the stimulation time. When new configuration data is set and the primary side attains the status of “Master”, the data will be transmitted to the secondary side. As shown in Figure 15b, for transmitting a “1”, the supply voltage of the PA maintains at V_D1_; but for transmitting a “0”, it will drop temporarily to V_D2_ as discussed in Section 4.2. A short period after the configuration data is transmitted the internal implant unit becomes the master and sends back the acknowledgement bit such that transmission of the configuration data will not be repeated.

Prior to current stimulation, power boosting is activated such that the transmission power is increased to deliver adequate power for the stimulation. As shown in Figure 15c, before the stimulation time, the Power Require bit P_R_ in the backscattered data is set to “0” to command the external control unit to increase the transmission power immediately.

The design parameters and measured data are summarized in Table 1. The primary coil has good quality factor (98), but that of the secondary coil is not as high (41). The inductance of the detection coil is around 300 nH, and the coupling factor with the primary coil is around 0.3 that achieves good sensitivity for backscattered data detection and does not have excessive interference on the primary coil. The 3-V LDO and the 1.2-V LDO have good power supply rejection ratio (PSRR) at both <1 kHz and 80 MHz such that the two ripple components are rejected. The total quiescent current of the secondary chip is around 40 μA. The maximum power transfer efficiency from the PA input to the rectifier output is around 8% when the distance of the two coils is 1 cm and rectifier loading current is 300 μA.

## 6. Conclusions

A TcES system that consists of an eye implant and an external component is proposed to help treating some eye diseases. The eye implant is wirelessly powered and controlled by the external component to generate the required bi-polar current pattern for stimulating the optic nerve. The current pattern can be controlled to have wide ranges of current amplitude, frequency and duty ratio. The power link and the bi-directional data link share the same pair of coils to reduce the overall size of the implant. Adaptive power delivery control method including power regulation and power boosting is proposed to cater for both coupling and load variations. Except for the secondary coil, the eye implant circuitry is wholly integrated on-chip and full functionality was demonstrated.

## Figures and Tables

**Table 1:** Measurement data.

**Primary coil**	Inductance	1 μH
	Q@40.68 MHz	98
**Secondary coil**	Inductance	170 nH
	Q@40.68 MHz	41
**Operational coil distance**	0–1.5 cm	
**3V low dropout regulator (LDO)**	Power supply rejection ratio (PSRR)	−40 dB@ < 1kHz
		−30 dB@80 MHz
**1.2V LDO**		−54 dB@ < 1 kHz
		−56 dB@80 MHz
**Total quiescent current of the secondary chip**	30 μA @light load–48 μA @1 mA heavy load	
**Maximum power transfer efficiency**	8% @ coil distance = 1 cm	
